# Molecular characterization of partial-open reading frames 1a and 2 of the human astroviruses in South Korea

**DOI:** 10.1186/1743-422X-7-221

**Published:** 2010-09-10

**Authors:** Jae in Lee, Gyu-Cheol Lee, Young hee Oh, Young ki Lee, Min young Kim, Chan Hee Lee

**Affiliations:** 1 Seoul Metropolitan Research Institute of Public Health & Environment, Gwacheon, Gyeonggi 427-070, Republic of Korea; 2 Water Analysis and Research Center, K-Water, Daejeon 306-711, Republic of Korea; 3 Department of Public Health, Graduate School of Public Health & Social Welfare, Dankook University, Cheonan 330-716, Republic of Korea; 4 Department of Microbiology, College of Natural Sciences, Chungbuk National University, Cheongju, Chungbuk 361-763, Republic of Korea

## Abstract

Human astroviruses (HAstVs) are among the major causes of gastroenteritis in South Korea. In this study, the partial regions of the open reading frame (ORF) 1a and ORF2 genes of HAstVs from gastroenteritis patients in nine hospitals were sequenced, and the molecular characterization of the viruses was revealed. 89 partial nucleotide sequences of ORF1a and 88 partial nucleotide sequences of ORF2 were amplified from 120 stool specimens. Phylogenetic analysis showed that most of the nucleotide sequences of ORF1a and ORF2 were grouped with HAstV type 1 but had evolutionary genetic distance compared with the reference sequences, such as the HAstV-1 prototype, Dresden strain, and Oxford strain. According to the phylogenetic analysis, some nucleotide sequences including SE0506041, SE0506043, and SE0506058, showed the discrepancy of the genotypes, but there was no proof of recombination among the HAstV types. In conclusion, this study showed that the dominant HAstV isolated from the Seoul metropolitan area in 2004-2005 was HAstV type 1, and that Korean HAstV-1 had the genetic distance in evolution compared with the reference sequences of HAstVs. Lots of nucleotide sequences of the ORF1a and ORF2 genes of HAstV will be useful for studying for the control and prevention of HAstV gastroenteritis in South Korea.

## Findings

Astroviruses (AstVs), belong to the *Astroviridae *family, are non-enveloped, single-stranded, and positive-sense RNA viruses [1]. Their genomes have both 5' and 3' non-translated regions, and contain three open reading frames (ORFs), denoted as ORF1a, ORF1b, and ORF2, which encode a serine protease, an RNA-dependent RNA polymerase, and a structural protein, respectively [1,2]. AstVs are known to infect humans as well as a variety of mammalian and avian species [3-5]. In humans, eight serotypes have been described, which have been associated with up to ~10% sporadic cases of nonbacterial diarrhea in children [6-10] and 0.5-15% outbreaks [11-13].

Walter et al. (2001) analyzed the gene of AstVs and found that the ORF2 region belonged to human AstV (HAstv)-5 whereas the ORF1b region belonged to HAstV-3, and that recombination occurred between the HAstV types [14]. Besides, in some other studies, recombination was found to occur between mamastroviruses and HAstV [15]. Such recombination may result in a new epidemic HAstV because it is similar to antigen drift of influenza viruses [16-19]. Therefore, characterization of HAstVs genome is important to understand the recombination between human and mammalian AstVs, the origin of the viruses, and their molecular evolution, as well as the phylogenetic relationship among the HAstV genotypes. For this purpose, there is a need to obtain more complete genome sequences of HAstV. The complete genome sequences of seven genotypes (HAstV-1, 2, 3, 4, 5, 6, and 8) and the HAstV-7 ORF2 sequence are available [18,20-23]. In this study, the partial nucleotide sequences of ORF1a and ORF2 of HAstVs, responsible for sporadic gastroenteritis in South Korea, were obtained, and their molecular characteristics were investigated.

From 2004 to 2005, stool specimens of patients suspected to have acute gastroenteritis were provided by nine hospitals located in the Seoul metropolitan area. 1 g of a stool specimen was added into 9 mL phosphate-buffered saline solution, and three or four 3-mm glass beads were added. The mixture was vigorously shaken via vortexing and was centrifuged at 4°C and 3000 rpm for 30 min. The 200 μL of 10% stool suspension was used for extracting RNA via the Tri-reagent method [24] and the extracted viral RNA was used for RT-PCR. The Mon340 and Mon348 primers were used for the amplification of the ORF1a region, and the Mon269 and Mon270 primers for the amplification of ORF2 (Table 1). For the synthesis of cDNA, 8 μL dNTP, 5 μL 5X buffer, 2.5 μL 10 pmole Mon348 or Mon270, 0.5 μL RNase inhibitor (Promega, Madison, WI), 0.5 μL MMLV reverse transcriptase (Promega), and 3.5 μL diethyl pyrocarbonate (DEPC) treated water and 5 μL RNA extract were added. The reaction conditions for the synthesis of cDNA were 42°C/60 min, 95°C/5 min, and 4°C soaking. For PCR, synthesized 5 μL cDNA was added to 6 μL dNTP, 5 μL 10× PCR buffer, each of the 2.5 μL 10 pmole primers, 0.5 μL exTaq polymerase (TaKaRa, Otsu, Shiga, Japan), and 28.5 μL DEPC treated water. The PCR conditions for ORF1a were 94°C/3 min, 94°C/30 sec, 50°C/20 sec, and 72°C/30 sec, 30 cycles, 72°C/5 min, and for ORF2, 94°C/3 min, 94°C/30 sec, 50°C/30 sec, and 72°C/1 min, 35 cycles, and 72°C/5 min. The amplified gene products were observed in 1.2% agarose gel. The PCR products were purified using a PCR purification kit (SolGent Co., Daejeon, South Korea) and were sequenced using ABI 3730XL DNA Analyzer (Applied Biosystems, Carlsbad, CA).

**Table 1:** Primers used for the detection of human astroviruses

Primers	Position*	Sequence (5'→3')	Size (bp)	References
Mon340	1182-1203	CGTCATTATTTGTTGTCATACT	289	[26]
Mon348	1450-1470	ACATGTGCTGCTGTTACTATG		

Mon269	4526-4545	CAACTCAGGAAACAGGGTGT	449	[24]
Mon270	4955-4974	TCAGATGCATTGTCATTGGT		

The nucleotide numbering is based on the sequences of human astrovirus type 1 (GenBank accession number: Z25771).

Multiple alignment and phylogenetic analysis were conducted using the ClustalX program and the PHYLIP package. For the distance matrix between the DNA sequences, the Dnadist program was used, and a phylogenetic tree was constructed using the neighbor-joining (NJ) method in the Neighbor program.

**Figure 1:**
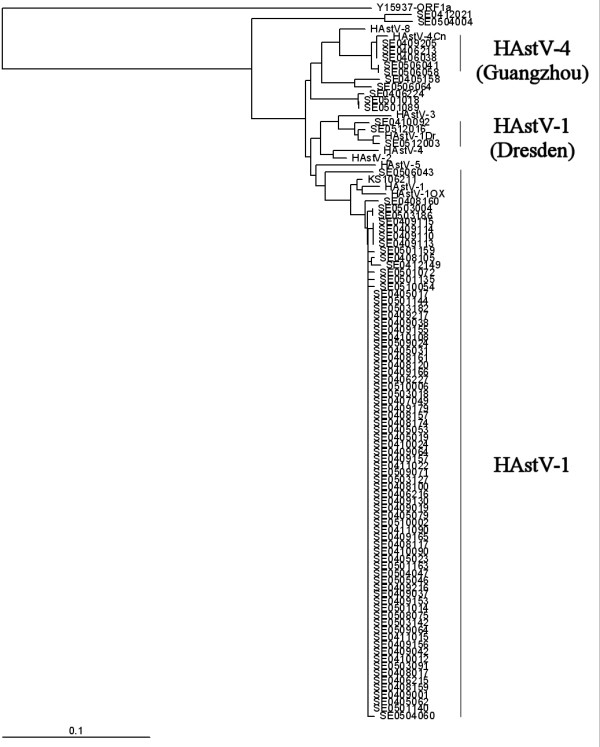
**Phylogenetic tree based on the partial sequences of open reading frame 1a amplified by the Mon340/348 primer pair**. The outgroup, the partial-open reading frame 1a nucleotide sequence of the sheep astrovirus, was selected from the nucleotide sequence of sheep astrovirus (GenBank accession number, Y15937).

**Figure 2:**
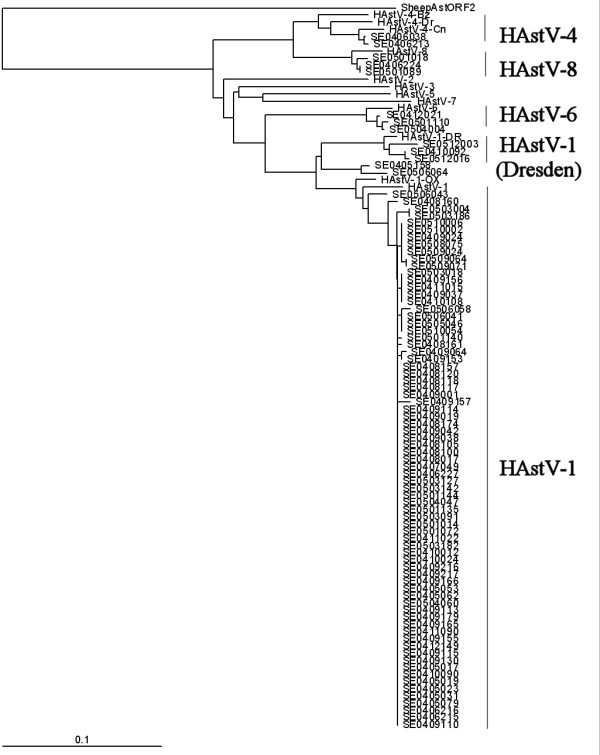
**Phylogenetic tree based on the partial sequences of open reading frame 2 amplified by the Mon269/270 primer pair**. The outgroup, the partial-open reading frame 2 nucleotide sequence of the sheep astrovirus, was selected from the nucleotide sequence of sheep astrovirus (GenBank accession number, Y15937).

In 89 of the 120 AstV specimens isolated from 2004 to 2005, the nucleotide sequence of the partial ORF1a amplicon amplified. The phylogenetic analysis results showed the nucleotide sequence of most of the partial ORF1as to be HAstV-1, and three isolates (SE0512016, SE0410092, and SE0512003) were grouped with the HAStV-1 Dresden strain (Fig. 1). The 73 HAstV-1 isolates were diverged earlier from sheep AstV, an outgroup, and were distant from the group to which the HAstV-1 prototype belonged, whereas the HAstV-1 prototype and the Oxford and KS106211 strains that were isolated in South Korea were grouped together (AF361036) [25] (Fig. 1). SE0506043 was placed between HAstV-1 and HAstV-5, and the phylogenetic branch diverged from HAstV-5 to the phylogenies of HAstV-2,4 (Goiania strain) and 3,1 (Dresden strain) and to the phylogeny of HAstV-8,4 (Guangzhou strain). SE0406224, SE0501018, SE0501089, SE0405158, and SE0506064 isolates diverged earlier and grouped together, keeping a distance from all the ten references. SE0412021 and SE0504004 were distant from all the references for which the nucleotide sequence of ORF1a was available (Fig. 1). SE0406038, SE0406213, SE0409205, SE0506041, and SE0506058 grouped with the HAstV-4 Guangzhou strain (Fig. 1). In case of ORF2, the 88 nucleotide sequences were analyzed and the phylogenetic tree was constructed. The HAstV-1 prototype, the Oxford strain, and the Dresden strain clustered, unlike in the case of ORF1, and the HAstV-4 Goiania, Dresden, and Guangzhou strains clustered in the same group (Fig. 2). In the nucleotide sequence of the ORF2 of the sheep AstV, which was closest to HAstV among the animal AstVs, HAstV-4 and 8 diverged earliest, followed by HAstV-3, 5, 7, 2, and 6 (Fig. 2). 75 partial ORF2 sequences were grouped in the place that diverged earlier than the HAstV-1 prototype, and the SE0405158 and SE0506064 isolates were in between the HAstV-1 Dresden isolates and the Oxford isolate whereas SE0512003, SE0512016, and SE0410092 belonged to the HAstV-1 Dresden isolate (Fig. 2). SE0501018, SE0501089, and SE0406224 grouped in the HAstV-8 reference, and SE0406038 and SE0406213 grouped in the HAstV-4 Guangzhou strain (Fig. 2). The SE0504004, SE0412021, and SE0501110 isolates grouped in HAstV-6, and no isolates grouped in HAstV-2, 3, 5, and 7.

For most of the isolates, all the nucleotide sequences of ORF1a and ORF2 belonged to HAstV-1 and were slightly distant from the references (the prototype and the Dresden and Oxford strains). The isolates, however, grouped together, with a high similarity between them. This indicates that the AstVs circulating in the Seoul metropolitan area were HAstV-1 and had the difference evolutionary course from the HAstV-1 circulating abroad. In several isolates, the genotypes of ORF1a and ORF2 did not coincide with each other. SE0506041 and SE0506058, however, which grouped in the HAstV-4 Guangzhou isolates in the analysis of the partial ORF1a, grouped in HAstV-1 in the analysis of the partial ORF2. In addition, SE0506043, which was in between HAstV-1 and HAstV-5 in the analysis of ORF1a, was closer to the prototype than HAstV-1 was. SE0406224, SE050018, and SE0501089, which were in between HAstV-8 and HAstV-1 in the analysis of ORF1a, grouped in places closer to HAstV-8 in the analysis of ORF2, and SE0405158 and SE0506064 were found to be HAstV-1, which was in between the HAstV-1 prototype and the Dresden strain.

Studies on the relation between the serotypes of HAstVs based on the base sequence of 300 nucleotides showed that there was a difference in genotypes between three ORFs [26]. Belliot et al. (1997) suggested that HAstV can be grouped into two genogroups, HAstV-1~-5 and HAstV-6~-7, based on ORF1a [25] and this was later supported by other studies [27,28]. In this study, all the references and isolates, excluding SE0504004, SE0510110, and SE0412021, also formed a large genogroup in the analysis of the partial ORF1a (Fig. 1). In contrast, Belliot et al. (1997) reported that such genotype was not found in their analysis of ORF1b and ORF2, and that HAstV could be classified into four clusters (HAstV-1; HAstV-6 and 2; HAstV-3, 4, and 8; and HAstV-5 and 7) in the analysis of the ORF2 partial sequence [26]. It has been reported, however, that in the analysis of a phylogenetic tree based on the full ORF2 amino acid sequence, three clusters (HastV-1, 7, and 3; HAstV-5 and 6; and HAstV-4 and 8) were found, and HAstV-2 was closer to the third cluster than to the other clusters [29]. In the analysis of the ORF2 partial sequences in this study, HAstV was classified into four clusters, as in the study by Belliot (1997) [26]. In the analysis of a phylogenetic tree based on the whole ORF2 sequence, however, HAstV could be classified into only three clusters, as in the study by Wang et al. (2001) [29]. Even if the genotype is well related with the serotype according to the partial sequence, a phylogenetic tree based on such relation may reflect a wrong phylogeny. Thus, it is considered that the evolutionary phylogeny of an AstV can be more accurately identified by a phylogenetic tree based on the whole base sequence of each gene. Although some studies asserted that the genotype discrepancy between the HAstV genes that occurred in their studies was due to the genetic recombinations between different serotypes [14,26], no proof of such recombination was found in any isolate that showed a discrepancy in genotypes. Although the mechanism of HAstVs' variations is not yet clear, the genetic variations by recombinations among HAsVs' types may evoke the appearance of new epidemic HAstVs, such as the influenza viruses, by antigenic drift.

## Competing interests

The authors declare that they have no competing interests.

## Authors' contributions

JIL, MYK and CHL conceived this study. JIL, YHO and YKL designed and conducted the experiments. JIL and GCL analyzed the sequence data and carried out the molecular phylogenetic analysis. JIL, GCL and CHL wrote the manuscript. All authors read and approved the final manuscript.

## References

[B1] Mendez E Arias CF Knipe DM, Howley PM Astroviruses Fields Virology 2007 I 5 Philadelphia: Lippincott Wiliams & Wilkins 981 1000

[B2] Finkbeiner SR Kirkwood CD Wang D Complete genome sequence of a highly divergent astrovirus isolated from a child with acute diarrhea Virol J 2008 5 117 18854035 10.1186/1743-422X-5-117 PMC2576171

[B3] Chu DK Poon LL Guan Y Peiris JS Novel astroviruses in insectivorous bats J Virol 2008 82 9107 9114 10.1128/JVI.00857-08 18550669 PMC2546893

[B4] Koci MD Schultz-Cherry S Avian astroviruses Avian Pathol 2002 31 213 227 10.1080/03079450220136521 12396344

[B5] Toffan A Jonassen CM De Battisti C Schiavon E Kofstad T Capua I Cattoli G Genetic characterization of a new astrovirus detected in dogs suffering from diarrhea Vet Microbiol 2009 139 147 152 10.1016/j.vetmic.2009.04.031 19477085 PMC7126621

[B6] Caracciolo S Minini C Colombrita D Foresti I Avolio M Tosti G Fiorentini S Caruso A Detection of sporadic cases of Norovirus infection in hospitalized children in Italy New Microbiol 2007 30 49 52 17319600

[B7] Glass RI Noel J Mitchell D Herrmann JE Blacklow NR Pickering LK Dennehy P Ruiz-Palacios G de Guerrero ML Monroe SS The changing epidemiology of astrovirus-associated gastroenteritis: a review Arch Virol Suppl 1996 12 287 300 9015126 10.1007/978-3-7091-6553-9_31

[B8] Kirkwood CD Clark R Bogdanovic-Sakran N Bishop RF A 5-year study of the prevalence and genetic diversity of human caliciviruses associated with sporadic cases of acute gastroenteritis in young children admitted to hospital in Melbourne, Australia (1998-2002) J Med Virol 2005 77 96 101 10.1002/jmv.20419 16032716

[B9] Klein EJ Boster DR Stapp JR Wells JG Qin X Clausen CR Swerdlow DL Braden CR Tarr PI Diarrhea Etiology in a Children's Hospital Emergency Department: A Prospective Cohort Study Clin Infect Dis 2006 43 807 813 10.1086/507335 16941358

[B10] Soares CC Maciel de Albuquerque MC Maranhao AG Rocha LN Ramirez ML Benati FJ Timenetsky Mdo C Santos N Astrovirus detection in sporadic cases of diarrhea among hospitalized and non-hospitalized children in Rio De Janeiro, Brazil, from 1998 to 2004 J Med Virol 2008 80 113 117 10.1002/jmv.21053 18041001

[B11] Akihara S Phan TG Nguyen TA Hansman G Okitsu S Ushijima H Existence of multiple outbreaks of viral gastroenteritis among infants in a day care center in Japan Arch Virol 2005 150 2061 2075 10.1007/s00705-005-0540-y 15841336

[B12] Lyman WH Walsh JF Kotch JB Weber DJ Gunn E Vinje J Prospective study of etiologic agents of acute gastroenteritis outbreaks in child care centers J Pediatr 2009 154 253 257 10.1016/j.jpeds.2008.07.057 18783794

[B13] Svraka S Duizer E Vennema H de Bruin E van der Veer B Dorresteijn B Koopmans M Etiological role of viruses in outbreaks of acute gastroenteritis in The Netherlands from 1994 through 2005 J Clin Microbiol 2007 45 1389 1394 10.1128/JCM.02305-06 17360839 PMC1865895

[B14] Walter JE Briggs J Guerrero ML Matson DO Pickering LK Ruiz-Palacios R Berke T Mitchell DK Molecular Characterization of a Novel Recombinant Strain of Human Astrovirus Associated with Gastroenteritis in Children Arch Virol 2001 146 2357 2367 10.1007/s007050170008 11811685 PMC7087139

[B15] Rivera R Nollens HH Venn-Watson S Gulland FM Wellehan JF Jr Characterization of phylogenetically diverse astroviruses of marine mammals J Gen Virol 2010 91 166 173 10.1099/vir.0.015222-0 19759240

[B16] Blackburne BP Hay AJ Goldstein RA Changing selective pressure during antigenic changes in human influenza H3 PLoS Pathog 2008 4 e1000058 10.1371/journal.ppat.1000058 18451985 PMC2323114

[B17] Guo L Gonzalez R Wang W Li Y Paranhos-Baccalà G Vernet G Wang J Complete genome sequence of human astrovirus genotype 6 Virol J 2010 7 29 10.1186/1743-422X-7-29 20137100 PMC2829535

[B18] Shen J Ma J Wang Q Evolutionary trends of A(H1N1) influenza virus hemagglutinin since 1918 PLoS One 2009 4 e7789 10.1371/journal.pone.0007789 19924230 PMC2773012

[B19] Tu ET Bull RA Greening GE Hewitt J Lyon MJ Marshall JA McIver CJ Rawlinson WD White PA Epidemics of gastroenteritis during 2006 were associated with the spread of norovirus GII.4 variants 2006a and 2006b Clin Infect Dis 2008 46 413 420 10.1086/525259 18177226

[B20] Jiang B Monroe SS Koonin EV Stine SE Glass RI RNA sequence of astrovirus: distinctive genomic organization and a putative retrovirus-like ribosomal frameshifting signal that directs the viral replicase synthesis Proc Natl Acad Sci USA 1993 90 10539 10543 10.1073/pnas.90.22.10539 8248142 PMC47812

[B21] Lewis TL Greenberg HB Herrmann JE Smith LS Matsui SM Analysis of astrovirus serotype 1 RNA, identification of the viral RNA-dependent RNA polymerase motif, and expression of a viral structural protein J Virol 1994 68 77 83 8254779 10.1128/jvi.68.1.77-83.1994 PMC236266

[B22] Oh D Schreier E Molecular characterization of human astroviruses in Germany Arch Virol 2001 146 443 455 10.1007/s007050170154 11338382

[B23] Silva PA Cardoso DD Schreier E Molecular characterization of human astroviruses isolated in Brazil, including the complete sequences of astrovirus genotypes 4 and 5 Arch Virol 2006 151 1405 1417 10.1007/s00705-005-0704-9 16421636

[B24] Noel JS Lee TW Kurtz JB Glass RI Monroe SS Typing of human astroviruses from clinical isolates by enzyme immunoassay and nucleotide sequencing J Clin Microbiol 1995 33 797 801 7790440 10.1128/jcm.33.4.797-801.1995 PMC228043

[B25] Kang YH Park YK Ahn JB Yeun JD Jee YM Identification of human astrovirus infections from stool samples with diarrhea in Korea Arch Virol 2002 147 1821 1827 10.1007/s00705-002-0844-0 12209320

[B26] Belliot G Laveran H Monroe SS Detection and genetic differentiation of human astroviruses, phylogenetic grouping varies by coding region Arch Virol 1997 142 1323 1334 10.1007/s007050050163 9267446

[B27] Gabbay YB Linhares AC Cavalcante-Pepino EL Nakamura LS Oliveira DS da Silva LD Mascarenhas JD Oliveira CS Monteiro TA Leite JP Prevalence of human astrovirus genotypes associated with acute gastroenteritis among children in Belém, Brazil J Med Virol 2007 79 530 538 10.1002/jmv.20813 17385695

[B28] Méndez-Toss M Griffin DD Calva J Contreras JF Puerto FI Mota F Guiscafré H Cedillo R Muñoz O Herrera I López S Arias CF Prevalence and genetic diversity of human astroviruses in Mexican children with symptomatic and asymptomatic infections J Clin Microbiol 2004 42 151 157 10.1128/JCM.42.1.151-157.2004 14715746 PMC321733

[B29] Wang QH Kakizawa J Wen LY Shimizu M Nishio O Fang ZY Ushijima H Genetic analysis of the capsid region of astroviruses J Med Virol 2001 64 245 255 10.1002/jmv.1043 11424111

